# Validation of Five Minimally Obstructive Methods to Estimate Physical Activity Energy Expenditure in Young Adults in Semi-Standardized Settings

**DOI:** 10.3390/s150306133

**Published:** 2015-03-13

**Authors:** Mikkel B. Schneller, Mogens T. Pedersen, Nidhi Gupta, Mette Aadahl, Andreas Holtermann

**Affiliations:** 1 Department of Nutrition, Exercise and Sports, University of Copenhagen, DK-2200 Copenhagen, Denmark; E-Mails: mkbs@steno.dk (M.B.S.); mtpedersen@nexs.ku.dk (M.T.P.); 2 National Research Centre for the Working Environment, DK-2100 Copenhagen, Denmark; E-Mail: ngu@nrcwe.dk; 3 Research Centre for Prevention and Health, the Capital Region of Denmark, Glostrup University Hospital, DK-2600 Glostrup, Denmark; E-Mail: metaad01@regionh.dk

**Keywords:** energy expenditure, accelerometry, activity type recognition, indirect calorimetry

## Abstract

We compared the accuracy of five objective methods, including two newly developed methods combining accelerometry and activity type recognition (Acti4), against indirect calorimetry, to estimate total energy expenditure (EE) of different activities in semi-standardized settings. Fourteen participants performed a standardized and semi-standardized protocol including seven daily life activity types, while having their EE measured by indirect calorimetry. Simultaneously, physical activity was quantified by an ActivPAL^3^, two ActiGraph GT3X+’s and an Actiheart. EE was estimated by the standard ActivPAL^3^ software (ActivPAL), ActiGraph GT3X+ (ActiGraph) and Actiheart (Actiheart), and by a combination of activity type recognition via Acti4 software and activity counts per minute (CPM) of either a hip- or thigh-worn ActiGraph GT3X+ (AGhip + Acti4 and AGthigh + Acti4). At group level, estimated physical activities EE by Actiheart (MSE = 2.05) and AGthigh + Acti4 (MSE = 0.25) were not significantly different from measured EE by indirect calorimetry, while significantly underestimated by ActiGraph, ActivPAL and AGhip + Acti4. AGthigh + Acti4 and Actiheart explained 77% and 45%, of the individual variations in measured physical activity EE by indirect calorimetry, respectively. This study concludes that combining accelerometer data from a thigh-worn ActiGraph GT3X+ with activity type recognition improved the accuracy of activity specific EE estimation against indirect calorimetry in semi-standardized settings compared to previously validated methods using CPM only.

## 1. Introduction

Physical inactivity is a major global risk factor for chronic diseases [1,2] and mortality [3]. Accordingly, a high level of physical activity reduces the risk of a range of chronic diseases and premature deaths [4,5]. The documentation of the health effects of physical activity is mainly based on studies using self-reported physical activity [6,7,8]. Self-reported physical activity has been shown to be imprecise and potentially biased, and may therefore not provide valid information on physical activity. Accordingly, objective measures of physical activity are recommended [9,10,11].

Physical activity is defined as “any bodily movement produced by skeletal muscles that results in energy expenditure (EE)” [12]. Thus, measurement of EE is the most frequently used objective approach for estimating physical activity. However, there are several challenges related to the measurement of physical activity EE [11,13,14]. Human EE is composed of resting EE, diet-induced thermogenesis, and physical activity EE [9,15]. Among these, physical activity is the main determinant of variations in total EE of a single individual in time [16], while physical activity and body size are the main determinants for differences in EE between individuals [13,17].

Indirect calorimetry has traditionally been used as a gold standard for assessing EE of physical activities. However, it is costly, relatively complex and inconvenient when measuring EE during free living conditions. Several cheaper, portable and convenient methods have been developed to measure physical activity EE in the field over several days. These methods include accelerometers, heart rate monitors, and a combination of the two. Ideally, these methods should be able to measure EE in free-living conditions during a period long enough to be representative of daily life, with no discomfort for the person or need for individual calibration, and at the same time provide information on intensity, duration, frequency and normal physical activity types and postures during daily living, like sitting, standing, lying down, walking and cycling [13]. Some very recent methods are very promising [18] but no currently available method fulfills all these criteria [9,10,19,20,21].

Methods using only accelerometers have been recommended in the assessment of EE due to their usability, relatively low cost, and ability to provide information on physical activity intensity, duration and frequency [4,20]. However, no prediction technique accurately estimates EE across all intensities [8]. Methods combining accelerometry and heart rate have tried to overcome this challenge, but are inaccurate in estimating individual EE [22,23,24]. The reason could be that these methods do not take physical activity type into account. It is well documented, that there is a significant relationship between physical activity intensity and EE within a specific physical activity type, but not between different physical activity types [13,25].

Several activity type classification models have been developed based on accelerometry [18,26,27,28]. One recent model provides information about physical activity type from two ActiGraph GT3X+ accelerometers placed on hip and thigh using the Acti4 software (National Research Center for the Working Environment, Copenhagen, Denmark; Federal Institute for Occupational Safety and Health (BAuA), Berlin, Germany) to recognize daily life activity types second-by-second [28]. Acti4 program has been shown to distinguish between the activity types sitting, standing, walking, running, cycling, and walking stairs with a sensitivity and specificity of approximately 99% [28]. Acti4 therefore offers accurate information about physical activity type [28], which can be combined with activity counts per minute (CPM) for estimation of physical activity EE. Thus, it would be of interest to investigate the accuracy of methods combining accelerometry and activity type recognition to determine EE of different activities. The purpose of this study was to compare the accuracy of EE estimation using two newly developed and three currently available and widely used methods against indirect calorimetry.

## 2. Experimental Section

### 2.1. Participants

Participants who were 20–40 years old and had a BMI < 30 kg/m^2^ were included in this study. 14 participants (eight women, six men) from Copenhagen and the surrounding area volunteered to participate in this study. All participants had previous experience of walking and running on a treadmill and cycling on an ergometer. The participants had no known pulmonary or cardiac diseases and did not take any prescription medication at the time of the study. The study was approved by the Ethics Committee for the Capital Region in Denmark (journal number H-2-2011-047) and conducted in accordance with the Helsinki declaration. All participants were informed about the risks and benefits of the study and gave their written consent to participate.

### 2.2. Study Design

The study followed a standardized and a semi-standardized protocol. The purpose of the standardized protocol was to establish linear correlation equations between CPM and EE in the different activity types. The purpose of the semi-standardized protocol was to compare the accuracy of EE estimation using two newly developed and three currently available methods against indirect calorimetry.

In the standardized protocol, the following physical activity types were performed in the given order: lying, sitting, standing, walking on treadmill at three different speeds (three bouts), running on treadmill at three different speeds, cycling on ergometer at three different speeds, and walking up/down one flight of stairs at three different speeds. Lying was performed for 12 min while all other bouts were performed for 5 min. All six bouts on treadmill (three walking bouts and three running bouts) were performed at 1% inclination [29]. Walking speeds were 4, 5 and 6 km/h respectively. Running speeds were 7, 8 and 9 km/h for women, and 7, 8.5 and 10 km/h for men. Cycling was performed on an ergometer with 70, 85 and 100 rpm and constant resistance of 0.016 kilopond × body mass for women and 0.02 kilopond × body mass for men. Walking stairs was performed on one flight of stairs (each step 0.173 m in vertical height) at 50, 70 and 90 steps/min respectively. Speeds of cycling and walking stairs were controlled by a cadence meter. For all activities except walking stairs, the last 2 min of each intensity were measured. For walking stairs, the CPM of the last 1 min 55 s at 50 steps/min, 1 min 50 s at 70 steps/min, and 2 min 8 s at 90 steps/min were averaged, to ensure an equal number of ascending and descending steps. From these measurement periods, linear regression equations were determined for each physical activity type based on average accelerometer CPM and the corresponding EE measured by indirect calorimetry.

The highest running speeds were calculated from the ACSM Metabolic Equations [30] to correspond to a metabolic requirement of 33.5 mL O_2_/kg/min for women and 36.8 mL O_2_/kg/min for men. The highest cycling resistance was calculated from ACSM Metabolic Equations to correspond to a metabolic requirement of 24 mL O_2_/kg/min for women and 28.6 mL O_2_/kg/min for men at 100 rpm. Intensities were selected so that the metabolic steady state could be reached by all participants at the highest intensity of each activity type, but still have wide difference between chosen intensities within each activity type. Based on this principle and differences in physical capacity between genders, we chose a higher range of resistance/speeds for men than for women for cycling and running. For stair climbing, the range was the same for both genders because of the lower metabolic requirement in order to reach steady state. All intensities of the activities aimed at resembling those performed in daily life activities.

In the semi-standardized protocol, each participant performed a 30 min semi-standardized protocol consisting of the same seven activity types as the standardized protocol. First, the activity types lying, sitting and standing were performed for 10 min (“stationary activity types”). Afterwards, the activities walking, running, cycling and walking stairs were performed for 20 min (“physical activity types”). The resistance of the cycle ergometer was set according to the procedure explained in the standardized protocol. The activity types were performed at self-selected intensities, duration and order, but with two restrictions; first, each period of each activity type should last at least 30 s to minimize time spent in transition states (generally 3–8 s) between activity types, which would not be classified as one of the activity types included in this study; second, each activity type should be performed at least twice to ensure significant contribution from all the included activity types.

### 2.3. Anthropometrics

Upon arrival, the height (Seca 222, Hamburg, Germany) and weight of all participants was measured in light clothing without shoes to the nearest 0.1 cm and 0.1 kg, respectively.

### 2.4. Energy Expenditure Measurements

For energy expenditure measurements, participants wore a COSMED K4b^2^ portable pulmonary gas exchange analyzer (COSMED Srl, Rome, Italy), an ActivPAL^3^ triaxial accelerometer (PAL Technologies Ltd., Glasgow, Scotland), an Actiheart combining uniaxial accelerometer and heart rate monitor (CamNtech, Cambridge, UK) and two ActiGraph GT3X+ triaxial accelerometers (ActiGraph TM, Pensacola, FL, USA) during the standardized and semi-standardized protocol.

COSMED K4b^2^ portable pulmonary gas exchange analyzer consisted of harness, battery, gas exchange analyzer, mask and mouthpiece and weighted ≈ 1.6 kg. The COSMED K4b^2^ software (version 9.0 b, COSMED Srl) calculated EE as a continuous summed value in kcal. COSMED K4b^2^ is shown to be a valid and reliable device to measure pulmonary gas exchange and thereby EE [31]. A reference gas calibration of the COSMED K4b^2^, a room air calibration and a gas delay calibration were performed before each test according to that described in the manual. A 3-liters syringe calibration of the flow meter was performed once a week. When a test was initiated, information on environmental temperature, humidity, height and weight (body mass +1.6 kg) of the participant was entered on the COSMED K4b^2^. After the test, the recorded total kilocalories (kcal/h) via COSMED K4b^2^ were transformed to kcal/kg/h (MET) by dividing the total kilocalories per hour of activity with the total weight of the participant and the COSMED K4b^2^.

### 2.5. Physical Activity Monitoring

The three different physical activity monitors utilized were (1) an ActivPAL^3^ triaxial accelerometer fixed by tape (3M, Hair-set, double sided adhesive tape, Oakdale, Minnesota, USA; Fixomull, BSN Medical, Hamburg, Germany; and Flexifix, Smith & Nephew, London, UK) at the right medial front of the thigh midway between the hip and knee joint with the x-axis pointing downwards; (2) an Actiheart combined uniaxial accelerometer in the vertical plane and the heart rate monitor with the main sensor attached to an electrocardiogram (ECG) electrode placed just below the apex of sternum and the secondary sensor fixed to an ECG electrode approximately 10 cm left to the main sensor at the same vertical height; (3) Two ActiGraph GT3X+ triaxial accelerometers fixed at the skin by tape (3 M, Hair-set, double sided adhesive tape; Fixomull, BSN Medical; and Flexifix, Smith & Nephew) at the right side of the hip near the upper point of the iliac crest with the x-axis pointing downwards, and at the right medial front of the thigh midway between the hip and knee joint with the x-axis pointing downwards [28]. Based on the mounted equipment, five different methods were utilized to estimate EE which are shown in Table 1.

**Table 1:** Methods based on mounted equipment to calculate energy expenditure of different activities.

Methods	Equipment and Software to Calculate Energy Expenditure
ActivPAL	ActivPAL^3^ accelerometer ActivPAL^3tm^ software *
ActiGraph	ActiGraph GT3X+ CPM from hip accelerometer the linear regression equation recommended by the ActiLife 6 software ^±^
Actiheart	Actiheart accelerometer’s CPM heart rate branched equation model recommended by Actiheart 4 software ^≠^
AGhip + Acti4	ActiGraph GT3X+ CPM from hip accelerometer activity type recognition from ActiGraph GT3X+ hip and thigh data with Acti4 software linear regressions established in the standardized protocol
AGthigh + Acti4	ActiGraph GT3X+ CPM from thigh accelerometer activity type recognition from ActiGraph GT3X+ hip and thigh data with Acti4 software linear regressions established in the standardized protocol

* ActivPAL^3tm^ process and presentation, v7.1.18, PAL Technologies Ltd., Glasgow, Scotland; ^±^ ActiLife v6.5.2, ActiGraph TM, Pensacola, FL, USA; ^≠^ Actiheart software version 4.0.100, CamNtech, Cambridge, UK.

### 2.6. Calculation of Estimated EE for ActivPAL

EE was calculated using the ActivPAL^3tm^ software. The software utilized default MET values of lying/sitting (1 MET), standing (1.4 MET), and for stepping (all physical activity), the default regression equation was utilized: MET = 0.0186 * steps/min + 1.4 [32]. For activity type classification, the ActivPAL^3tm^ software was used to recognize the activity types lying/sitting, standing and stepping.

### 2.7. Calculation of Estimated EE for Actiheart

Accelerometry was measured at 32 Hz and heart rate was collected as inter-beat-intervals. Data was transformed to an estimated EE using the branched equation parameters of “Group Cal JAP2007” incorporated in the Actiheart 4 software (Actiheart software version 4.0.100, CamNtech, Cambridge, UK). Actiheart data was downloaded and pre-processed using the commercial software provided by Actiheart 4. Actiheart data processing were done according to the “MRC Epidemiology Unit Physical Activity Data Processing Guidelines” [33,34]. The raw heart rate signals contain noise and un-physiological heart rate measures, which needed to be removed and corrected. Data were checked for missing heart rate data by the Actiheart software according to previously recommended guidelines [34]. Sleeping heart rate was required by the Actiheart software to estimate EE, but no measurement of a full sleep cycle was made. Instead sleeping heart rate was predicted as the lowest measured 1 min epoch of resting heart rate during the 12 min of lying in the standardized protocol −8%, as heart rate has been found to drop from 64 at consciousness to 59 at stage 4 sleep, corresponding to ~8% lower sleeping heart rate than resting heart rate [35,36].

### 2.8. Calculation of Estimated EE for ActiGraph

The hip mounted ActiGraph GT3X+ accelerometer were set to measure at a sampling rate of 60 Hz and EE was calculated using the algorithm “Crouter Adult (2012)” [37,38] in the ActiLife6 software.

### 2.9. Calculation of Estimated EE for AGhip + Acti4 and AGthigh + Acti4

Estimation of EE for AGhip + Acti4 and AGthigh + Acti4 was based on activity type recognition by Acti4, which is described in Section 2.10. Using the leave-one-subject-out approach, separate fixed values of EE for lying, sitting and standing were estimated for each participant based on corresponding measured EE by indirect calorimetry in the standardized protocol. These fixed values were then used to estimate EE of lying, sitting and standing for the methods AGhip + Acti4 and AGthigh + Acti4 in the semi-standardized protocol. For the activity types walking, running, cycling and walking stairs, CPM from the hip and thigh worn ActiGraph GT3X+ in the standardized protocol were plotted against EE obtained from indirect calorimetry to determine linear regression equations between activity type and intensity. For this purpose, CPM were calculated for each 5 min activity period minus the first and last 30 s in the different activity types and intensities performed. Due to different resistances in cycling between women and men, a linear regression equation was modeled for women and men separately. ActiLife6 calculated CPM in 10 s epochs and second-by-second Acti4 output was generated from the semi-standardized protocol for activity type recognition. The dominant activity type for each 10 s epoch was determined by Acti4 and EE of each activity type was calculated from average measured CPM and the linear regression equation found in the standardized protocol for the corresponding activity type. Acti4 contains the activity type category “move”, which is a miscellaneous category for physical activity types that are not recognized as walking, running, cycling, or walking stairs [28]. Energy expenditure of epochs dominated by the “move” category was calculated from the linear regressions equations of walking. The sum of EE from all 10 s epochs in the semi-standardized protocol were calculated for each participant and converted to MET.

### 2.10. Activity Classification

Acti4 is a recently developed MatLab (MathWorks Inc., Natick, MA, USA) program shown to provide valid physical activity type classification based on raw acceleration output measured by ActiGraph GT3X+ accelerometer [28,39]. Acti4 uses a simple algorithm classification tree based on raw accelerations, standard deviation, inclination and forward/backward angle intervals to determine activity type. Using the Acti4, different sedentary and physical activities were determined according to the procedure explained in Skotte, Korshoj, Kristiansen, Hanisch and Holtermann [28] study. Acti4 has been shown to distinguish between the activity types lying, sitting, standing, walking, running, cycling, and walking stairs with a sensitivity and specificity of approximately 99% with two ActiGraph GT3X+ placed on the hip and thigh [28]. For further information on Acti4, please refer to Skotte, Korshoj, Kristiansen, Hanisch and Holtermann [28].

### 2.11. Statistical Analysis

Linear regression was used to develop prediction models for EE in the different activity types using the variable CPM. The leave-one-subject-out cross-validation method was used in linear correlation equations for all activities including walking, running, cycling and walking stairs. The correlation between CPM and EE was tested for significance using Pearson’s coefficient of determination.

Paired *t* tests were used to analyze differences in EE measured by COSMED K4b^2^ and different methods during various activities in the standardized protocol and during first 10 min of stationary activity types, last 20 min of physical activity types and total 30 min in the semi-standardized protocol. Additionally, besides using results of paired *t* tests, we also calculated mean square error (MSE) to assess the accuracy of the five methods against indirect calorimetry. The MSE was calculated using following formula:

(1) $$\text{MSE}=\frac{N-1}{N}s_{i}^{2}+{\left({\bar{z}}_{i}\right)}^{2}$$

where, *N* = number of participants, s*_i_* is the estimated standard deviation of the differences between two methods, and ${\bar{z}}_{i}$ is the average mean difference between two methods.

Additionally, accuracy of EE estimation was further assessed by Pearson coefficient of determination, intra-class correlations, and Bland-Altman plots. Bland-Altman plots were created from measured EE by COSMED K4b^2^ plotted against the difference between estimated EE of the analyzed method and EE measured by COSMED K4b^2^. The limits of agreement in the Bland-Altman plots were set as mean difference ± 1.96 SD.

The statistical software IBM SPSS Statistics version 20 (IBM Corp., New York, NY, USA) was used for statistical analysis. The significance level was set to *p* < 0.05.

## 3. Results

### 3.1. Descriptive Results

**Figure 1:**
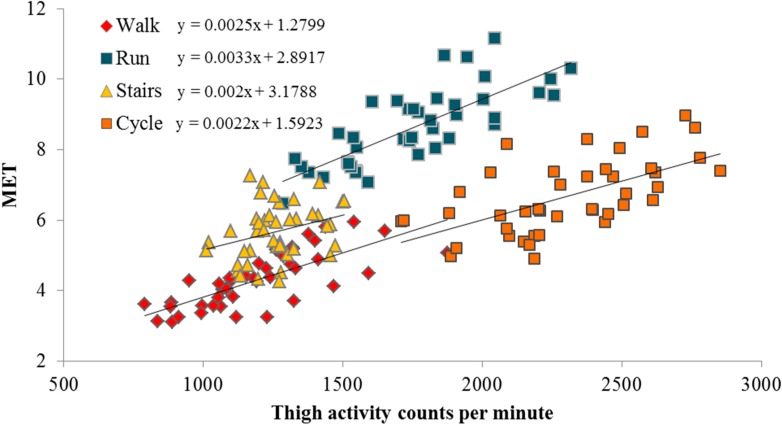
Scatter plots and linear regression equations between CPM of a thigh-mounted ActiGraph GT3X+ accelerometer and EE measured by indirect calorimetry of physical activity types.

Table 2 shows anthropometric data of the participants. Energy expenditure values for 28 of 210 periods estimated by Actiheart in the standardized protocol were discarded due to missing heart rate data. Figure 1 shows an example of the developed linear regressions from the standardized protocol. The corresponding resultant linear regressions using Acti4+thigh and Acti4+hip method are shown below:
WalkingActi4 + hipMET = 0.004 CPM + 2.003, *R*² = 0.64, SEE = 0.48Acti4 + thighMET = 0.003 CPM + 1.282, *R*² = 0.53, SEE = 0.53RunningActi4 + hipMET = 0.002 CPM +4.914, *R*² = 0.32, SEE = 0.92Acti4 + thighMET = 0.003 CPM + 2.889, *R*² = 0.61, SEE = 0.69CyclingActi4 + hipMET = 0.003 CPM + 0.670 gender (1 = female, 2 = male) + 5.293, *R*^2^ = 0.26, SEE = 0.91Acti4 + thighMET = 0.003 CPM + 1.094 gender (1 = female, 2 = male) − 1.132, *R*^2^ = 0.63, SEE = 0.64Walking stairsActi4 + hipMET =0 .003 CPM + 3.669, *R*² = 0.41, SEE = 0.61Acti4 + thighMET = 0.002 CPM + 3.187, *R*² = 0.09, SEE = 0.76

**Table 2:** Characteristics of the participants.

Variables	Total (*n* = 14)	Women (*n* = 8)	Men (*n* = 6)
Age (years)	27.7 ± 3.3	27.9 ± 4.6	27.6 ± 1.1
Height (cm)	172.3 ± 9.2	165.5 ± 6.9	180.0 ± 3.6
Weight (kg)	68.2 ± 8.4	63.3 ± 7.6	73.8 ± 5.5
BMI (kg/m^2^)	22.9 ± 1.4	23.0 ± 1.2	22.8 ± 1.7

Data as Mean ± SD. BMI, Body mass index.

A significant correlation between CPM and EE was found for all activity types, except for the hip-worn ActiGraph GT3X+ in cycling for men. Based on Pearson coefficient of determination (*R*^2^), CPM of the hip- and thigh-worn ActiGraph GT3X+ explained 64% and 56% respectively of the variance in EE in walking, 32% (*p* < 0.01) and 61% (*p* < 0.01) in running, 9% (*p* = 0.23) and 47% (*p* < 0.01) in cycling for men, 36% (*p* < 0.01) and 70% (*p* < 0.01) in cycling for women, and 41% (*p* < 0.01) and 9% (*p* < 0.05), for walking stairs.

### 3.2. EE Estimation in Standardized Settings

The accuracy of the different methods compared to COSMED K4b^2^ to estimate EE in different activity types are shown in Table 3.

**Table 3:** Comparisons between measured EE by COSMED K4b^2^ (indirect calorimetry) and estimated EE by different methods for different activities performed during a standardized protocol.

	COSMED K4b^2^	ActivPAL	ActiGraph	Actiheart	AGhip + Acti4	AGthigh + Acti4
Lying	1.27 ± 0.20	1.25 ± 0.00 (0.04)	1.00 ±0.00 (0.11) *	1.25 ± 0.14 (0.05)	1.27 ± 0.02 (0.04)	1.27 ± 0.02 (0.04)
Sitting	1.16 ± 0.10	1.25 ± 0.00 (0.02) *	1.00 ± 0.00 (0.04) *	1.32 ± 0.16 (0.06) *	1.16 ± 0.01 (0.01)	1.16 ± 0.01 (0.01)
Standing	1.30 ± 0.11	1.40 ± 0.00 (0.02) *	1.00 ± 0.00 (0.10) *	1.97 ± 0.75 (1.08) *	1.30 ± 0.01 (0.01)	1.30 ± 0.01 (0.01)
Walking 1	3.47 ± 0.26	3.65 ± 0.13 (0.10)	3.02 ± 0.23 (0.32) *	3.90 ± 0.74 (0.75)	3.70 ± 0.40 (0.22)	3.83 ± 0.42 (0.34) *
Walking 2	4.24 ± 0.23	3.87 ± 0.11 (0.17) *	3.59 ± 0.30 (0.57) *	4.80 ± 0.84 (0.97)	4.27 ± 0.42 (0.19)	4.29 ± 0.52 (0.23)
Walking 3	5.22 ± 0.42	4.06 ± 0.12 (1.48) *	4.26 ± 0.39 (1.20) *	5.78 ± 1.06 (1.29)	4.93 ± 0.45 (0.37)	4.88 ± 0.59 (0.51)
Running 1	7.61 ± 0.68	4.60 ± 0.13 (9.57) *	7.34 ± 1.57 (2.33)	9.51 ± 1.59 (4.84) *	8.23 ± 0.61 (0.96) *	7.73 ± 0.33 (0.40)
Running 2	8.67 ± 0.77	4.46 ± 0.24 (18.31) *	8.75 ± 1.76 (2.62)	10.71± 1.58 (5.01) *	8.76 ± 0.56 (0.58)	8.65 ± 0.36 (0.49)
Running 3	9.62 ± 0.77	4.41 ± 0.30 (27.58) *	9.37 ± 1.90 (3.29)	11.35 ± 1.85 (4.28) *	9.00 ± 0.54 (1.02) *	9.52 ± 0.60 (0.56)
Cycling 1	5.67 ± 0.46	4.09 ± 0.08 (2.67) *	1.01 ± 0.02 (21.89) *	4.64 ± 1.05 (2.01) *	6.57 ± 0.33 (0.93) *	6.10 ± 0.46 (0.53) *
Cycling 2	6.64 ± 0.59	4.64 ± 0.08 (4.32) *	1.14 ± 0.31 (30.52) *	6.83 ± 2.33 (4.95)	6.72 ± 0.43 (0.25)	6.71 ± 0.49 (0.40)
Cycling 3	7.69 ± 0.79	4.85 ± 0.06 (8.67) *	1.38 ± 0.59 (40.50) *	8.59 ± 2.60 (6.52)	6.91 ± 0.55 (1.15) *	7.25 ± 0.58 (0.75)
Stairs 1	4.88 ± 0.44	2.55 ± 0.11 (5.66) *	2.93 ± 0.17 (4.06) *	5.87 ± 1.91 (4.06)	5.13 ± 0.34 (0.44)	5.48 ± 0.22 (0.69) *
Stairs 2	5.63 ± 0.39	2.99 ± 0.15 (7.14) *	3.51 ± 0.17 (4.74) *	6.90 ± 2.17 (5.71) *	5.70 ± 0.28 (0.29)	5.75 ± 0.26 (0.32)
Stairs 3	6.50 ± 0.45	3.29 ± 0.05(10.51) *	4.09 ± 0.31 (6.20) *	7.98 ± 2.35 (7.06) *	6.16 ± 0.31 (0.50)	5.80 ± 0.26 (0.86) *

Results are shown as MET ± SD (MSE). * = *p* < 0.05 for MET compared to COSMED.

During stationary activities such as lying, sitting and standing, ActiGraph was the most inaccurate method in predicting the EE compared to COSMED followed by Actiheart. For example, Actiheart showed a 52% overestimation (MSE = 1.08) in EE in standing compared to indirect calorimetry (*p* < 0.01).

During different physical activities, ActivPAL significantly underestimated EE of walking 4 and 5 km/h, and all running, cycling and walking stairs intensities. ActiGraph significantly underestimated EE at all walking, cycling and walking stairs intensities, but estimated running EE accurately. Actiheart mostly overestimated EE of all physical activity type intensities (except for cycling at 70 rpm) with significant results for 6 of the 12 physical activity type intensities. Increments in cycling EE from 70 → 85 → 100 rpm was larger for Actiheart (4.64 → 6.83 → 8.59 MET) and smaller for AGhip+Acti4 (6.57 → 6.72 → 6.91 MET) compared to COSMED K4b^2^ (5.67 → 6.64 → 7.69 MET). During cycling, the CPM of the Actiheart accelerometer was below the cut point of 25 CPM set in the branched equation used to estimate EE by the Actiheart4 software in 90.2% of measures at 70 rpm, 66.1% at 85 rpm and 27.7% at 100 rpm. Although cycling EE estimated via AGthigh+Acti4 was significantly different from COSMED at one of the intensities (70 rpm), this method overall predicted the EE of cycling with lowest error of the tested methods. Increments in walking stairs EE from 50 → 70 → 90 steps/minute was smaller for AGthigh+Acti4 (5.48 → 5.75 → 5.80 MET) compared to COSMED K4b^2^ (4.88 → 5.63 → 6.50 MET).

We compared our fixed EE values (MET) measured using COSMED with those in the Physical Activity Compendium [40]. The standard values in the Physical Activity Compendium for lying quietly, sitting quietly in general, and standing quietly are 1.3, 1.3, 1.3 MET, respectively. These values are similar to those measured via COSMED (lying = 1.3 MET, sitting = 1.2 MET, standing =1.3 MET) in our study.

### 3.3. EE Estimation in Semi-Standardized Settings

EE estimation of all methods in the semi-standardized protocol is shown in Table 4. In the stationary activity types of the semi-standardized protocol, Actiheart overestimated EE by 45% (*p* < 0.01, *R*^2^ = 0.02, ICC = −0.04) compared to COSMED K4b^2^. The methods ActivPAL, ActiGraph, AGhip + Acti4 and AGthigh + Acti4 underestimated stationary activity EE by 13% (*p* < 0.01, *R*^2^ = 0.17, ICC = −0.09), 17% (*p* < 0.01, *R*^2^ = 0.00, ICC = −0.03), 10% (*p* < 0.01, *R*^2^ = 0.15, ICC = −0.30), and 12% (*p* < 0.05, *R*^2^ = 0.13, ICC = −0.27) respectively, compared to COSMED K4b^2^.

**Table 4:** Comparisons of EE of stationary and physical activities measured by COSMED K4b^2^ (indirect calorimetry) and estimated by different methods during a semi-standardized protocol.

Activities	COSMED K4b^2^	ActivPAL	ActiGraph	Actiheart	AGhip + Acti4	AGthigh + Acti4
Stationary	1.49 ± 0.03	1.30 ± 0.00(0.05) *	1.24 ± 0.02(0.08) *	2.16 ± 0.18(0.91) *	1.34 ± 0.02(0.04) *	1.31 ± 0.02(0.04) *
Physical	7.34 ± 0.25	3.91 ± 0.03(12.49) *	5.58 ± 0.28(3.67) *	7.48 ± 0.52(2.05)	6.25 ± 0.13(1.60) *	6.61 ± 0.20(0.25)
Total	5.39 ± 0.17	3.04 ± 0.02(5.84) *	4.13 ± 0.19(1.83) *	5.69 ± 0.39(1.39)	4.59 ± 0.09(0.80) *	5.31 ± 0.13(0.12)

Results are shown as MET ± SD (MSE). * = *p* < 0.05 for MET compared to COSMED.

In the physical activity types of the semi-standardized protocol (last 20 min), ActivPAL underestimated EE by 47% (*p* < 0.01, *R*^2^ = 0.14), ActiGraph by 24% (*p* < 0.01, *R*^2^ = 0.49), and AGhip+Acti4 by15% (*p* < 0.01, *R*^2^ = 0.68, ICC = 0.67) compared to COSMED K4b^2^. Actiheart and AGthigh+Acti4 were not significantly different from COSMED K4b^2^ for assessing EE during physical activity types in the semi-standardized protocol at group level. However, AGthigh + Acti4 (MSE = 0.25, *R*^2^ = 0.77, ICC = 0.87) explained more of the individual variation in measured physical activity EE by COSMED K4b^2^ than Actiheart (MSE = 2.05, *R*^2^ = 0.45, ICC = 0.52).

### 3.4. Estimation of EE of Individuals

**Figure 2:**
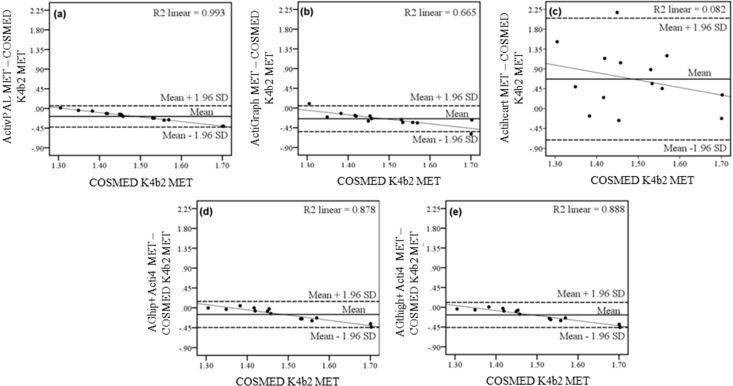
Bland-Altman plots of the EE estimated in the stationary activity types lying, sitting and standing in the semi-standardized protocol of the methods (**a**) ActivPAL; (**b**) ActiGraph; (**c**) Actiheart; (**d**) AGhip+Acti4 and (**e**): AGthigh+Acti4. Dashed lines represent the limits of agreement (±1.96 SD), full line the mean difference between estimated and measured EE. The thin line represents the linear correlation plot of the individual scatter plots and *R*^2^-value (*R*^2^) the Pearson coefficient of determination of individual scatter plots to this linear correlation line.

In the stationary activity types of the semi-standardized protocol, the Bland-Altman plots, linking measured EE to the difference between measured EE and calculated EE of the different sensors, showed a high coefficient of determination for the methods ActivPAL (*R*^2^ = 0.99), ActiGraph (*R*^2^ = 0.67), AGhip + Acti4 (*R*^2^ = 0.88) and AGthigh + Acti4 (*R*^2^ = 0.89) This indicates a systematic bias of underestimating EE with increasing EE. Actiheart showed wide limits of agreement but low coefficient of determination between individual scatter plots (*R*^2^ = 0.08) in the stationary activity types (Figure 2c) implying no systematic bias.

**Figure 3:**
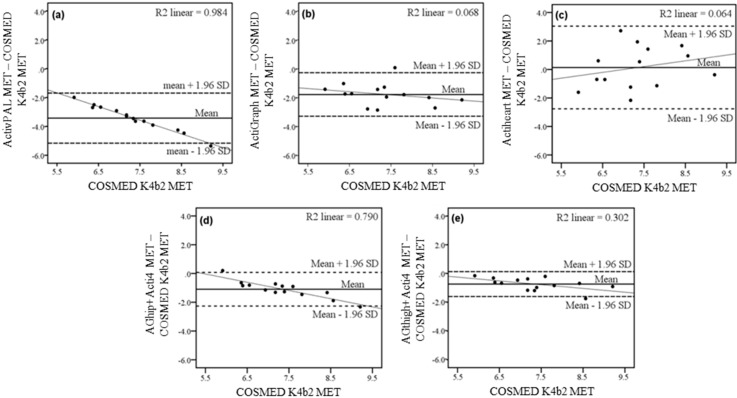
Bland-Altman plots of the EE estimated in the physical activity types walking, running, cycling and walking stairs in the semi-standardized protocol of the methods: (**a**) ActivPAL; (**b**) ActiGraph; (**c**) Actiheart; (**d**) AGhip + Acti4 and (**e**): AGthigh + Acti4. Dashed lines represent the limits of agreement (±1.96 SD), solid line represents the mean difference between estimated and measured EE. The thin line represents the linear regression between EE by COSMED and difference between EE estimated by different methods and COSMED. *R*^2^ is the Pearson coefficient of determination of individual scatter plots to this linear regression line.

In the physical activity types of the semi-standardized protocol, the Bland-Altman plot in Figure 3a showed high coefficient of determination between EE measured via COSMED K4b^2^ and the difference between EE measured using ActivPAL and COSMED K4b^2^ (*R*^2^ = 0.98). ActivPAL showed systematic bias of underestimating EE with higher measured EE by COSMED K4b^2^. There was a low coefficient of determination (*R*^2^ = 0.07) but wide limits of agreement between measured EE and the difference between measured EE and predicted EE by ActiGraph (Figure 3b). The limits of agreement between measured EE and the difference between measured and predicted EE by the tested methods were visually wider for Actiheart (Figure 3c) than both AGhip + Acti4 (Figure 3d) and AGthigh + Acti4 (Figure 3e). Actiheart showed large variation in the EE measurements compared to COSMED K4b^2^ as shown in the Bland-Altman plot, but low coefficient of determination(*R*^2^ = 0.06, Figure 3c) indicating no systematic bias. 13 of the 14 participants had their EE underestimated by the AGhip + Acti4 method compared to COSMED K4b^2^ (Figure 3d). AGhip + Acti4 showed a systematic bias of underestimating EE with increasing measured EE by COSMED K4b^2^ (*R*^2^ = 0.79). However, for the link between measured EE and the difference between measured EE and EE predicted by AGthigh + Acti4, a downward sloping trend was seen with lower coefficient of determination (*R*^2^ = 0.30) than other methods except Actiheart (Figure 3e).

## 4. Discussion

The results of this study showed that combining accelerometry with activity type recognition in the AGthigh + Acti4 method improved the accuracy of physical activity EE estimation at group and individual level in a standardized and semi-standardized protocol, compared to three previously used and validated methods.

### 4.1. Estimation of Energy Expenditure during Stationary Activities

The results showed that the Actiheart method, which uses combined accelerometry and heart rate data, largely overestimated EE of the stationary activity types in the semi-standardized protocol with large individual variations shown by the MSE value of 0.91 compared to MSE ranging from 0.04 to 0.08 for the other methods. This finding is in line with a study by Crouter, Churilla and Bassett [22] in which standing EE was found to be significantly overestimated by Actiheart data and a branched equation and showed a higher SD, compared to indirect calorimetry [22]. This result indicates that the Actiheart method has a limited ability to estimate EE during lying, sitting and standing.

The results of our study also showed that the fixed value approach (an activity type or group of activity types are given a fixed MET) for EE estimation used by the methods ActivPAL, ActiGraph, AGhip + Acti4 and AGthigh + Acti4, was better than the combined method utilized by Actiheart for estimating EE of stationary activities. However, the fixed value approach was biased by individual variation, as seen by the increasingly underestimated EE by the methods ActivPAL, ActiGraph, AGhip + Acti4 and AGthigh + Acti4 with higher measured EE (Table 3). In daily life, an individual utilizes the majority of energy in stationary activities [41], and even small differences between estimated and actual EE can result in highly inaccurate estimates of EE in free-living conditions. Accuracy could be increased by recognizing stationary activity types and ascribing a fixed value to each activity type and dividing the population into subgroups depending on personal characteristics such as age, sex, height and weight, which were not included in this study.

### 4.2. Estimation of EE during Physical Activity

The accuracy of AGthigh + Acti4 and Actiheart was better than ActivPAL, ActiGraph and AGhip + Acti4 for estimating physical activity EE at group level in the semi-standardized protocol. Additionally, the AGthigh + Acti4 (MSE = 0.25) method had a lower MSE and explained 32% more of the individual variation in physical activity EE in the semi-standardized protocol than Actiheart (MSE = 2.05) compared to COSMED K4b^2^. This shows that AGthigh + Acti4 accurately estimated physical activity EE with only minor errors for individuals. AGhip + Acti4 numerically underestimated mean physical activity EE, but explained individual variations well. Actiheart accurately estimated physical activity EE at group level, but lacked accuracy in assessing individual variations, a finding in agreement with previous studies [10,22,24,42].

The large individual variations seen for Actiheart may be caused by several potential sources of error. The heart rate/EE relationship is highly individualistic [14,15]. Poor accelerometer assessment of movement can be due to its placement on the chest and its uniaxial and low quality accelerometer [23,43]. The hierarchy of the branched equation can lead to an undesirable weighting of CPM and heart rate in the estimation of EE, as for cycling in this study. Actiheart showed significant overestimation of EE compared to COSMED K4b^2^ in nine of 12 bouts of physical activity performed in the standardized protocol, but was not significantly different in the physical activity types in semi-standardized conditions at group level. In the semi-standardized protocol, EE was estimated continuously over a range of activity types and intensities, while activity type and intensity was fixed with steady state heart rate reached in the standardized protocol. ActivPAL underestimated EE of walking at 6 km/h and all running, cycling and walking stairs intensities compared to COSMED K4b^2^ in the standardized protocol. This indicates that the step cadence approach used by ActivPAL was not applicable for estimating physical activity EE during these activities. ActiGraph only estimated running EE accurately, while walking, cycling and walking stairs EE was underestimated due to the activity types’ different relationships between CPM and EE. The developed linear regression equations between CPM and EE for AGhip + Acti4 and AGthigh + Acti4 accurately estimated EE at the middle intensity of all physical activity types performed in the standardized protocol. The generally lower increments in EE with increasing physical activity intensity was probably due to linear regression equations being relatively too little influenced by CPM and too much influenced by the y-intercept. This is a problem as y-intercept value should approach resting EE to contain the best physiological intercept [44]. In this study, all participants ran at higher velocities when self-chosen in the semi-standardized protocol than in the standardized protocol. Running EE may have been gradually underestimated at intensities over 8.5 km/h by AGhip + Acti4 (y-intercept = 4.92 MET) but not by AGthigh + Acti4 (y-intercept = 2.89 MET), due to their y-intercepts. A combination of the differences in linear equations for running and the running intensities performed in the semi-standardized protocol are the likely explanations of why AGthigh + Acti4 was more accurate than AGhip + Acti4 in estimating physical activity EE. A better correlation between CPM and EE could enhance the accuracy of physical activity EE estimation in two ways. First, it would lead to CPM explaining individual variations in EE more accurately across a wide range of intensities of a physical activity type. Second, the y-intercept value would better explain the metabolic cost of the activity type, rather than the metabolic cost of performing the middle intensity of the protocol from which the correlation equations were based on. This shows a need to increase the correlation of linear correlations across physical activity types in order to improve the accuracy of the methods AGhip + Acti4 and AGthigh + Acti4.

Significant linear regression equations were found between CPM and EE measured by COSMED K4b^2^ for AGthigh + Acti4. Similar results were obtained for AGhip + Acti4, except in cycling among men. This resulted in only minor differences in estimated EE between cycling intensities performed in standardized settings by AGhip + Acti4 compared to COSMED K4b^2^ (AGhip + Acti4 ~ 2%, COSMED K4b^2^ ~ 15%). The accelerometer part of the chest-worn Actiheart measured an activity value (X) < 25 CPM in 90.2% of measures at cycling 70 rpm, 66.1% at 85 rpm and 27.7% at 100 rpm during cycling. The Actiheart user manual states that the activity value X = 25 CPM was chosen to be low enough to be exceeded in cycling [33], but this was not observed in the majority of measures in this study. These results showed that measured accelerations of a thigh-worn accelerometer was correlated to EE in cycling, while both a hip- and chest-worn accelerometer were not. Estimation of cycling EE in daily life activities has generally not been addressed in the literature. However, cycling ought to be included as a daily life activity type, especially in countries with high prevalence of daily cycling, like Denmark and Holland.

The systematic error depicted by a visual inspection of the Bland-Altman plots in Figure 3 showed that ActivPAL, ActiGraph, AGhip + Acti4 and AGthigh + Acti4 generally underestimated the physical activity EE with increasing intensity. However, this systematic error was lower when estimating EE with ActiGraph and AGthigh + Acti4 compared to ActivPAL and AGhip + Acti4. Actiheart did not show a clear systematic bias of under- or overestimation of EE with increasing EE, but Actiheart did show a large individual variation. As ActiGraph generally underestimated EE, the Acti4 + thigh method was the most preferable of these methods for estimating physical activity EE, specifically during activities with higher intensities.

### 4.3. Activity Type Recognition

The combination of activity type recognition and accelerometry improved the accuracy of estimated EE compared to estimations using only CPM in this study, as found in several other studies [10,13,41]. Estimation of walking stairs EE by AGthigh + Acti4 was accurate at 70 steps/min, but almost no difference was seen between walking stairs 70 steps/min and walking stairs 50 steps/min or walking stairs 90 steps/min (AGthigh + Acti4 ~ 3%, COSMED K4b^2^ ~ 15%, Figure 3e). This showed that CPM based linear equationfor a thigh-worn ActiGraph GT3X+ accelerometer was not a good predictor of changes in walking stairs intensity in this study. Energy expenditure was almost exclusively determined by the recognition of walking stairs as activity type. Despite this, no significant differences were found between walking stairs EE by AGthigh+Acti4 and COSMED K4b^2^. This finding is in agreement with a previous study of Bonomi, Plasqui, Goris and Westerterp [13] who found that identification of activity types performed without assessing intensity improved estimation of free-living EE compared to only CPM. Recognition of activity type can add characteristics of a movement pattern to a relationship between CPM and EE, e.g., the contribution to EE from the ascending/descending movement pattern of walking stairs. In this manner, information on activity type can account for differences in the correlation between accelerometer movement and EE among activity types [41].

Individual contributions from different activity types to daily life EE is highly variable, and no single existing regression model is able to accurately estimate physical activity EE over a range of activity types at different intensities [45]. Several pattern recognition systems, like Acti4, are now capable of improving EE estimation by recognizing activity types with high sensitivity and specificity [18,27,28,45,46].

### 4.4. Limitations, Strengths, Future Perspectives and Implication of the Results

A limitation of this study was the inclusion of a homogenous study population in terms of BMI and age, which limits the generalization of the findings to other populations. Additionally we utilized a small sample size of 14 participants. A third limitation was the lack of inclusion of an array of daily life activities, such as gardening and vacuuming. Thus, validation of our method in identifying a wider range of lifestyle activities and predicting their EE should be conducted in future studies using a larger sample size. A fourth limitation is that we utilized laboratory based activity types such as running on the treadmill and stationary bicycling, which does not completely imitate daily life running and cycling. A validation of our newly developed method in free living conditions is therefore recommended. A fourth limitation was the range of intensities included in the standardized protocol. Running 7 km/h and walking stairs 50 steps/min were slow and perceived as unnatural by some participants. Future research is needed in order to find the best relationship between CPM and EE in each activity type across a wide range of intensities covering those seen in daily life. A fifth limitation of the study is that we utilized leave-one-subject-out cross-validation method and not two separate groups to develop and validate the regression equations. However, leave-one-subject-out cross-validation method has been shown to be a valid validation technique and has been used by many previous studies [47,48]. Another limitation of the study is that the activities performed in the semi-standardized protocol were not performed in pre-determined order, which may generate some variance, but it can also be considered a strength since it better represents conditions of daily life.

The main strength of this study was the simultaneous comparison of the ability of newly developed methods and existing and widely used methods for estimating EE during different physical activities. Another strength was the utilization of an accurate method, Acti4, which predicts various stationary and physical activities with very high sensitivity and specificity. This study recommends using AGthigh + Acti4 method, compared to other methods, in estimating the EE of various activities such as sitting, lying, standing, walking, running, cycling and walking stairs. The advantages of this method are its accuracy compared to other methods and feasibility as it requires subject to wear only one accelerometer, if you combine lying and sitting into one activity type.

We recommend future studies to establish correlation equations between CPM of a thigh-placed triaxial accelerometer and measured EE in different physical activity types with a larger and more heterogeneous population (especially with respect to age and BMI), and a broader range of intensities and daily life physical activities, e.g., gardening, vacuuming. New methods to estimate EE frequently appears, e.g., a machine learning approach found to increase the accuracy of estimated EE of sedentary, moderate and vigorous physical activity time compared to existing ActiLife6 algorithms [49], and these new methods should be validated directly against each other in the same free-living conditions.

## 5. Conclusions

This study showed that combining data from a thigh-worn accelerometer with activity type recognition in the newly developed method AGthigh + Acti4 improved accuracy of physical activity EE estimation in semi-standardized conditions, compared to commonly used and validated methods not taking activity type into account.
